# Mechanisms of pathogenicity and the quest for genetic modifiers of kidney disease in branchiootorenal syndrome

**DOI:** 10.1093/ckj/sfad260

**Published:** 2023-10-13

**Authors:** Sebastian Sewerin, Charlotte Aurnhammer, Cene Skubic, Kaja Blagotinšek Cokan, Jera Jeruc, Damjana Rozman, Frederick Pfister, Katalin Dittrich, Brigitte Mayer, Ria Schönauer, Friederike Petzold, Jan Halbritter

**Affiliations:** Division of Nephrology, University of Leipzig Medical Center, Leipzig, Germany; Current affiliation: Broad Institute of MIT and Harvard, Cambridge, MA, USA; Division of Nephrology, University of Leipzig Medical Center, Leipzig, Germany; Institute of Biochemistry, Centre for Functional Genomics and Bio-Chips, Faculty of Medicine, University of Ljubljana, Ljubljana, Slovenia; Institute of Biochemistry, Centre for Functional Genomics and Bio-Chips, Faculty of Medicine, University of Ljubljana, Ljubljana, Slovenia; Institute of Pathology, Faculty of Medicine, University of Ljubljana, Ljubljana, Slovenia; Institute of Biochemistry, Centre for Functional Genomics and Bio-Chips, Faculty of Medicine, University of Ljubljana, Ljubljana, Slovenia; Institute of Pathology, University of Erlangen Medical Center, Erlangen, Germany; Current affiliation: Humanpathologie Dr. med. Manfred Weiß MVZ GmbH, Erlangen-Tennenlohe, Germany; Division of Pediatric Nephrology, University of Leipzig Medical Center, Leipzig, Germany; Division of Pediatric Nephrology, University of Dresden Medical Center, Dresden, Germany; Division of Nephrology, University of Leipzig Medical Center, Leipzig, Germany; Current affiliation: Department of Nephrology and Medical Intensive Care, Charité Universitätsmedizin Berlin, Berlin, Germany; Division of Nephrology, University of Leipzig Medical Center, Leipzig, Germany; Division of Nephrology, University of Leipzig Medical Center, Leipzig, Germany; Current affiliation: Department of Nephrology and Medical Intensive Care, Charité Universitätsmedizin Berlin, Berlin, Germany

**Keywords:** branchiootorenal syndrome, chronic kidney disease, modifier genes, patient-derived fibroblasts, phenotypic heterogeneity

## Abstract

**Backgound:**

Branchiootorenal (BOR) syndrome is an autosomal dominant disorder caused by pathogenic *EYA1* variants and clinically characterized by auricular malformations with hearing loss, branchial arch anomalies, and congenital anomalies of the kidney and urinary tract. BOR phenotypes are highly variable and heterogenous. While random monoallelic expression is assumed to explain this phenotypic heterogeneity, the potential role of modifier genes has not yet been explored.

**Methods:**

Through thorough phenotyping and exome sequencing, we studied one family with disease presentation in at least four generations in both clinical and genetic terms. Functional investigation of the single associated *EYA1* variant c.1698+1G>A included splice site analysis and assessment of EYA1 distribution in patient-derived fibroblasts. The candidate modifier gene *CYP51A1* was evaluated by histopathological analysis of murine *Cyp51^+/^^−^* and *Cyp51^−/−^* kidneys. As the gene encodes the enzyme lanosterol 14α-demethylase, we assessed sterol intermediates in patient blood samples as well.

**Results:**

The *EYA1* variant c.1698+1G>A resulted in functional deletion of the EYA domain by exon skipping. The EYA domain mediates protein-protein interactions between EYA1 and co-regulators of transcription. EYA1 abundance was reduced in the nuclear compartment of patient-derived fibroblasts, suggesting impaired nuclear translocation of these protein complexes. Within the affected family, renal phenotypes spanned from normal kidney function in adulthood to chronic kidney failure in infancy. By analyzing exome sequencing data for variants that potentially play roles as genetic modifiers, we identified a canonical splice site alteration in *CYP51A1* as the strongest candidate variant.

**Conclusion:**

In this study, we demonstrate pathogenicity of *EYA1* c.1698+1G>A, propose a mechanism for dysfunction of mutant EYA1, and conjecture *CYP51A1* as a potential genetic modifier of renal involvement in BOR syndrome.

## INTRODUCTION

Pathogenic variants in the *EYA1* gene are the major cause of the dominantly inherited branchiootorenal (BOR) syndrome (MIM #113650) which is characterized by auricular malformations with associated hearing loss, branchial arch anomalies, and congenital anomalies of the kidney and urinary tract (CAKUT) [1]. Less frequently, patients with BOR syndrome present facial dysmorphism and skeletal malformations [2]. These phenotypic traits exhibit highly variable and incomplete expression profiles between and within families [3]. Even amongst related family members, renal involvement in BOR syndrome can range from organ agenesis to normal kidney function [3].

Random monallelic expression, characterized by random silencing of one allele during embryonic development, is proposed to underlie the phenotypic variability [4]. Depending on the kidney's developmental stage, about 50% of the metanephric mesenchyme exhibit monoallelic expression [5].

Modifier genes represent yet another opportunity to explain the phenotypic heterogeneity in BOR syndrome [6]. We hypothesized that modifier genes might affect EYA1 function through aberrant protein-protein interactions or dysregulations of associated signaling pathways.

For murine kidney development, the *EYA1* gene is indispensable: Heterozygous *EYA1* deficient mice exhibit renal abnormalities and conductive hearing loss similar to those observed in BOR syndrome, whereas mice with a homozygous knock-out of *EYA1* lack ears and kidneys altogether, the latter being attributed to an absence of ureteric bud outgrowth and a subsequent failure of metanephric mesenchyme induction [7].

In this study, we define the molecular basis of BOR syndrome in a family segregating a previously reported heterozygous canonical splice site variant in *EYA1* [8]. In light of the broad spectrum of renal involvement in this family, ranging from normal kidney function in adulthood to end-stage kidney disease in infancy, we embark on identifying genetic modifiers of renal disease in BOR syndrome.

## MATERIALS AND METHODS

### Study participants and their phenotypic characterization

Informed consent was obtained from all subjects involved in the study. Clinical evaluation of the index family included physical examination, laboratory testing, renal ultrasound, and, in one adult subject, audiometry testing.

### Genetic analysis

For genetic analysis, written informed consent was obtained from family members III-1 and III-2 who also consented in their role as legal representatives for their children IV-1 and IV-2 (Institutional Review Board IRB00001750, University of Leipzig; #402/16-ek). DNA was isolated from peripheral blood cells from both parents and their affected children. Exome sequencing (ES) data were obtained and analyzed using both an unbiased and a candidate gene approach.

### Derivation of primary dermal fibroblasts and cell culture

A skin biopsy was obtained from patient III-1 after he had given written informed consent. The tissue sample was immediately submerged in media, i.e., Dulbecco's Modified Eagle Medium (#41966052, Gibco, Thermo Fisher Scientific, Waltham, MA, USA) with 10% v/v FBS Superior (#S0615, Biochrom/Merck Millipore, Berlin, Germany) and 1% v/v penicillin/streptomycin (#15140122, Gibco, Thermo Fisher Scientific, Waltham, MA, USA). Macroscopically visible fat was dissected from the skin tissue which was then cut into several pieces. These were seeded in T flasks such that they adhered to the surface and were incubated in a 5% CO_2_ atmosphere at 37°C until outgrowth of primary cells was observed.

### EYA1 splice site analysis

RNA was extracted from pelleted primary dermal fibroblasts from patient III-1 using the RNeasy Plus Mini Kit (#74134, Qiagen, Hilden, Germany). Genomic DNA was removed with ezDNase Enzyme before RNA was reverse transcribed into cDNA with the SuperScript IV VILO Master Mix (#11766050, Invitrogen, Thermo Fisher Scientific, Waltham, MA, USA). For splice site analysis, PCR primers were chosen to span a 290 bp amplicon extending from the transcribed exon 16 (forward primer 5′-CCCAGCATTGGCGAAAGTCCTGC-3′) through the 3′ untranslated region (reverse primer 5′-CTGTGCGCTGTCAAAGTGCCGAG-3′) of *EYA1* mRNA (NM_000503.6). Upon agarose gel electrophoresis, PCR products from patient fibroblast cDNA separated into three distinct bands, henceforth referred to as lower, middle, and upper bands, respectively. Following gel extraction and reamplification of PCR products, the extracted lower and middle bands exhibited rather clean single banding on an agarose gel. The corresponding reamplified DNA samples were thus subjected to Sanger sequencing. To obtain a clean isolate of the upper DNA fragment, its reamplified PCR product was again subjected to agarose gel electrophoresis; DNA was extracted from the desired (upper) band, followed by another reamplification step and Sanger sequencing. However, unambiguous sequencing results for this band could not be obtained (see also the caption of Fig. 2C). RNA from dermal fibroblasts of a control patient with no known pathogenic *EYA1* variant was extracted, reverse transcribed, amplified, and sequenced separately. The *Taq* PCR Core Kit (#201225, Qiagen, Hilden, Germany) was used for all PCRs and the GenUP PCR/Gel Cleanup Kit (#BR0700503, biotechrabbit, Berlin, Germany) for all gel extractions.

### Immunoblot analysis

Immunoblotting was performed on whole cell lysates from primary dermal fibroblast cultures.

Pelleted cells were resuspended in RIPA buffer (#89901, Thermo Scientific, Thermo Fisher Scientific, Waltham, MA, USA) with protease inhibitor cocktail (#P8340, Sigma-Aldrich, St. Louis, MO, USA) added. With intermittent mixing, the suspension was incubated on ice for a total of 30 min. After centrifugation for 30 min at 4°C, the supernatant was collected. Protein concentrations were determined with the Pierce BCA Protein Assay Kit (#23227, Thermo Scientific, Thermo Fisher Scientific, Waltham, MA, USA).

Proteins were separated by electrophoresis on a NuPAGE 3–8% Tris-Acetate gel (#EA0378BOX, Invitrogen, Thermo Fisher Scientific, Waltham, MA, USA) in NuPAGE Tris-Acetate SDS Running Buffer (#LA0041, Invitrogen, Thermo Fisher Scientific, Waltham, MA, USA) and blotted with the iBlot 2 system (#NW0412AIB2, Invitrogen, Thermo Fisher Scientific, Waltham, MA, USA). The membrane was cut at the 85 kDa marker. Following blocking in 3% milk-TBST for 1 h, the membrane pieces were incubated with primary antibodies in blocking solution overnight at 4°C. The next day, the membrane pieces were washed three times with TBST for 5 min each before they were incubated with secondary antibody in TBST for 1 h at room temperature and washed again three times with TBST for 5 min each. For antibody signal detection, the WesternBright ECL kit (#K-12045-D50, Advansta, San Jose, CA, USA) was used. The following primary antibodies were used: EYA1 rabbit polyclonal antibody (#22658-1-AP, Proteintech, Rosemont, IL, USA) at 1:500 and recombinant anti-vinculin antibody (#ab129002, Abcam, Cambridge, UK) produced in rabbit at 1:10 000. Anti-rabbit IgG, HRP-linked antibody (#7074, Cell Signaling Technology, Danvers, MA, USA) was used as secondary antibody at 1:2 000.

### Immunofluorescence analysis

Primary dermal fibrobasts were grown to confluency and seeded at 1:1 in a chambered coverslip (#80826, ibidi, Gräfelfing, Germany). The following day, cells were washed with PBS and fixed for 15 min. After two washes with PBS, cells were permeabilized for 15 min. Following another two PBS washes, cells were blocked for 1–1.5 h before they were incubated with primary antibody (EYA1 rabbit polyclonal antibody, #22658-1-AP, Proteintech, Rosemont, IL, USA) in PBS at 1:50 for 3 h at room temperature. Cells were then washed three times with PBS and incubated with secondary antibody (Alexa Fluor 555 goat anti-rabbit IgG (H + L), #A21428, Invitrogen, Thermo Fisher Scientific, Waltham, MA, USA) in PBS at 1:200 for 1.5 h at room temperature. After another three PBS washes, nuclei were counterstained with DAPI (#R37606, Invitrogen, Thermo Fisher Scientific, Waltham, MA, USA) in PBS for 10 min at room temperature. Cells were then washed with PBS and mounted in ibidi Mounting Medium (#50001, ibidi, Gräfelfing, Germany). For image acquisition, we used an Axio Observer.Z1 microscope with an ApoTome.2 imaging system (Carl Zeiss Microscopy, Jena, Germany). Images were processed with ZEN 2 software (Carl Zeiss Microscopy, Jena, Germany). For quantitative analysis, nuclear fluorescence intensities were determined with ImageJ software [9].

### Quantitative real-time PCR (qPCR) analysis

RNA from dermal fibroblasts was reverse transcribed into cDNA with the SuperScript IV VILO Master Mix with ezDNase Enzyme (#11766050, Invitrogen, Thermo Fisher Scientific, Waltham, MA, USA). For qPCR on patient and control cDNA, we used the Biozym Blue S'Green qPCR Kit Separate ROX kit (#331416S, Biozym Scientific, Hessisch Oldendorf, Germany) with primers specific for human *EYA1* and 18S rRNA (housekeeping primers) on a QuantStudio 3 system (Thermo Fisher Scientific, Waltham, MA, USA).

### Analysis of cholesterol biosynthesis intermediates

Seven main cholesterol intermediates (zymosterol, testis meiosis-activating sterol, lanosterol, 24-dehydrolathosterol, desmosterol, zymostenol, and lathosterol) were quantitatively analyzed in the sera of three family members (III-1, III-2, IV-1) and two adult control subjects (one male, one female) by means of a gas chromatographic/mass spectrometric method [10]. Blood samples had been taken after a fasting period.

### Histopathological analysis of kidneys from heterozygous *Cyp51* knock-out mice as well as heterozygous and homozygous *Cyp51* knock-out mouse embryos

Heterozygous and homozygous *Cyp51* knock-out mice were generated as B6;129SV^Cyp51tm1Bfro^ (*Cyp51^+/^^−^, Cyp51^−^^/^^−^*) mice and processed as previously reported [11, 12]. Total kidney weights were determined in 16 weeks old *Cyp51^+/^^−^* mice and matched controls. Histopathological analysis was performed on PAS-stained sections of paraffin embedded kidneys from 16 weeks old female (*n* = 2) and male (*n* = 2) *Cyp51^+/^^−^* mice as well as on those of sex matched controls (*n* = 2; one sample per sex). The number of glomerular generations per kidney section was counted and the sizes of ten randomly selected glomeruli were measured and compared. Furthermore, tissue sections were graded for glomerulosclerosis, interstitial fibrosis/tubular atrophy, inflammation, immature parenchyma, cysts, and metaplastic tissue. Histopathological analysis was also performed on PAS-stained sections of paraffin embedded kidneys from *Cyp51^+/^^−^* and *Cyp51^−^^/^^−^* knock-out mouse embryos (E14.5).

### Three-dimensional EYA domain (ED) protein model

The model showing the C-terminal ED (amino acid positions 322–592) of EYA1 protein was obtained from the AlphaFold database [13, 14] (entry AF-Q99502-F1) and custom colored using the UCSF Chimera software [15].

### Statistical analyses

Statistical analyses were performed as indicated using Prism 9 (GraphPad Software, Boston, MA, USA) with *p* < 0.05 considered statistically significant.

## RESULTS

### Clinical characterization of a family with BOR syndrome reveals marked phenotypic heterogeneity

**Figure 1:**
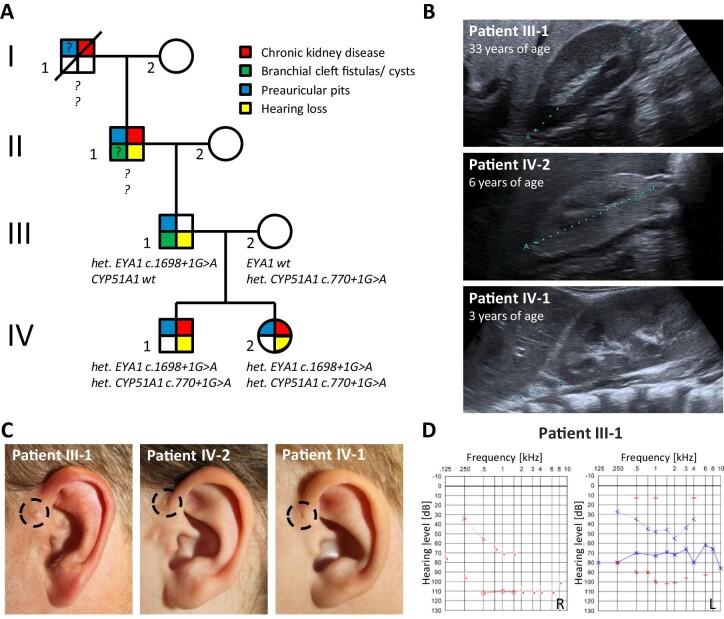
Pedigree and clinical characteristics of the index family. (A) Pedigree. (B) Ultrasonographic images of right kidneys from patient III-1 (A 82.6 mm), patient IV-2 (A 44.7 mm), and patient IV-1 (A 19.5 mm). Note the prominent kidney allograft next to the hypotrophic and dysplastic right kidney in patient IV-1. (C) Close-up images of left ears from indicated patients with preauricular pits highlighted by dashed circles. (D) Audiograms from patient III-1 showing functional deafness of the right ear and combined sensorineural hearing loss on the left side. Red color, right ear; blue color, left ear; downward arrow, no response; circle, air conduction of the right ear; arrow pointing right, bone conduction of the right ear; cross, air conduction of the left ear; arrow pointing left, bone conduction of the left ear; dash, contralaterally masked air conduction; zig zag, contralaterally masked bone conduction.

To gain insights into the mechanism driving the phenotypes associated with BOR syndrome, we clinically characterized a family of European descent with at least four generations of affected family members. These patients exhibited preauricular pits and developed hearing loss as well as, to varying extents, chronic kidney disease (CKD), indicating BOR syndrome (Table 1, Fig. 1A–D). These phenotypic traits segregated in an autosomal dominant fashion (Fig. 1A). Of note, patient III-1 is known to have had branchial fistulae which were surgically removed in childhood.

**Table 1:** Genetic variants, results from kidney ultrasonography, and kidney function parameters from patients III-1 and III-2 as well as their children IV-1 and IV-2.

	III-1 (father)	III-2 (mother)	IV-1 (son)	IV-2 (daughter)
Genetic variants	*EYA1* c.1698+1G>A; *CYP51A1* wt	*EYA1* wt; *CYP51A1* c.770+1G>A	*EYA1* c.1698+1G>A; *CYP51A1* c.770+1G>A	*EYA1* c.1698+1G>A; *CYP51A1* c.770+1G>A
Age (years)	35	31	2	6
Kidney ultrasound; kidney length (mm)	normal	normal; R 106 mm, L 113	bilateral dysplasia; R 26, L 36	bilateral hypotrophic dysplasia; R 45, L 50
Hemoglobin (mmol/l)	8.9	8.1	7.3	7.6
Serum creatinine (mg/dl)	1.1	0.6	3.98	1.0
Cystatin C (mg/l)			5.38	1.75
eGFR (CKD-EPI/Schwartz) (ml/min/1.73 m^2^)	92	120	11	62.4
Blood urea nitrogen (BUN) (mmol/l)	5.0	3.6	27.3	9.8
Proteinuria (protein to creatinine ratio; mg/g)	no	no	2900	532
Albuminuria (albumin to creatinine ratio; mg/g)	no	no	2300	233

Reference ranges: hemoglobin 7.5–9.9 mmol/l; serum creatinine 0.67–1.2 mg/dl; cystatin C 0.5–0.95 mg/l; eGFR >90 ml/min/1.73 m^2^, blood urea nitrogen <8.3 mmol/l, proteinuria <70 mg/g, albuminuria <20 mg/g. R, right; L, left; wt, wild-type.

The grandfather (II-1), the father (III-1), and both children (IV-1 and IV-2) developed hearing loss. Patient II-1 has been showing hearing impairment since the age of 40 years. His son, patient III-1, developed functional deafness of the right ear following right ear surgery in childhood and suffers from a combined sensorineural hearing loss on the left side (60–80 dB with a conductive component of 15–50 dB), requiring a hearing aid for his left ear (Fig. 1D). The son (IV-1) of patient III-1 presented with conductive hearing loss at the age of two years, requiring multiple tympanic drainages, while his daughter (IV-2) was provided with hearing aids at age ten years because of sensorineural hearing impairment following a SARS-CoV2 infection with acute hearing loss.

Kidney ultrasonography of participating family members demonstrated a wide range of structural alterations that resulted in different stages of CKD (Fig. 1B). While patient III-1 presented with morphologically normal kidneys and unimpaired organ function (serum creatinine 1.1 mg/dl, eGFR 92 ml/min/1.73 m^2^, no proteinuria), his son (IV-1) developed chronic kidney failure (serum creatinine 3.98 mg/dl, cystatin C 5.38 mg/l, eGFR 11 ml/min/1.73 m^2^, protein to creatinine ratio 2.9 g/g, albumin to creatinine ratio 2.3 g/g) at age two years due to bilateral kidney dysplasia and underwent preemptive living kidney transplantation with the organ donated from his mother. Of note, patient IV-1 had had a low birth weight (2610 g on 39 + 3 weeks of pregnancy, second percentile) and required supplementary feeding as he failed to thrive. His sister (IV-2) developed CKD G2A2 (serum creatinine 1.0 mg/dl, cystatin C 1.75 mg/l, eGFR 62.4 ml/min/1.73 m^2^, protein to creatinine ratio 532 mg/g, albumin to creatinine ratio 233 mg/g) at six years of age, with hypotrophic and dysplastic kidneys on ultrasonography. Both grandfather (II-1) and great-grandfather (I-1) were also reported to have suffered from CKD. However, the etiologies of their renal diseases could not exactly be determined. The mother (III-2) presented with normal kidney function (serum creatinine 0.6 mg/dl, eGFR 120 ml/min/1.73 m^2^, no proteinuria).

### ES reveals heterozygous splice site variant in EYA1

**Figure 2:**
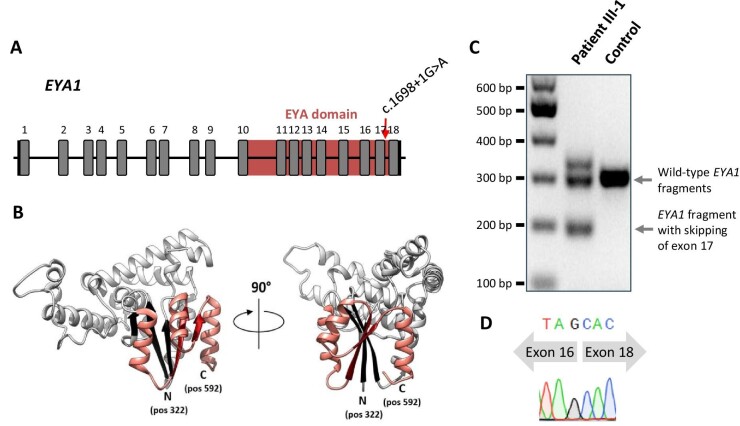
Analysis of the *EYA1* splice site affected by c.1698+1G>A. (A) Gene structure and location of canonical splice site mutation c.1698+1G>A in *EYA1* (NM_000503.5) with transcription factor binding EYA domain formed by amino acids at positions 322–592. (B) Three-dimensional structure of EYA1 protein (PDB ID Q99502) with indicated N- and C-terminal ends of its EYA domain. The sequence preserved in the patients is depicted in light grey (including three strands from the beta sheet structure (black)), whereas the sequence part that is aberrant in the patients is shown in salmon (including two strands from the beta sheet structure (red)). (C) Agarose gel image, showing separation of mutant from wild-type *EYA1* cDNA fragments. The analysis is based on a 290 bp amplicon spanning the cDNA region from the transcribed exon 16 through the 3′ untranslated region. The transcript corresponding to the lower band in the middle lane lacks exon 17. That corresponding to the upper band seems to lack exon 17, too, while also containing additional nucleotides, probably as an artifact from PCR. The cDNA was derived from primary dermal fibroblasts. (D) Chromatogram from Sanger sequencing the lower band evident in the middle lane in (C), showing the skip of exon 17.

In patient III-1 and both of his children (IV-1 and IV-2), ES revealed the *EYA1* splice site variant c.1698+1G>A (NM 000503.5) located in intron 17 close to a donor splice site. In silico, this variant was predicted to compromise proper splice site function (MaxEnt: −100.0%, NNSPLICE: −100.0%) (Fig. 2A). The variant is absent from the gnomAD database (v2.1.1) and had been reported previously in another family with BOR syndrome [8]. Subject III-2 who is not affected by BOR syndrome harbored wild-type *EYA1*.

### *In vitro* characterization of the EYA1 variant c.1698+1G>A reveals major alterations of the ED and impaired nuclear translocation of EYA1 protein

In order to illuminate how *EYA1* c.1698+1G>A causes dysfunction of EYA1 protein, we investigated the impact of the altered splice donor site on the respective mRNAs. To this end, we probed cDNA from primary dermal fibroblasts from patient III-1 for the presence of a 290 bp mRNA fragment specific to wild-type *EYA1*. This analysis yielded a mutant transcript version, exhibiting the lack of exon 17 (Fig. 2C and D; Fig. S1A, see online supplementary material) as well as the single nucleotide variant c.1755T>C (Fig. S1A, see online supplementary material). The out-of-frame deletion is predicted to drastically alter the C-terminal portion of the ED protein sequence (Fig. S1B, see online supplementary material) which is highly conserved across species (Fig. S1C, see online supplementary material).

**Figure 3:**
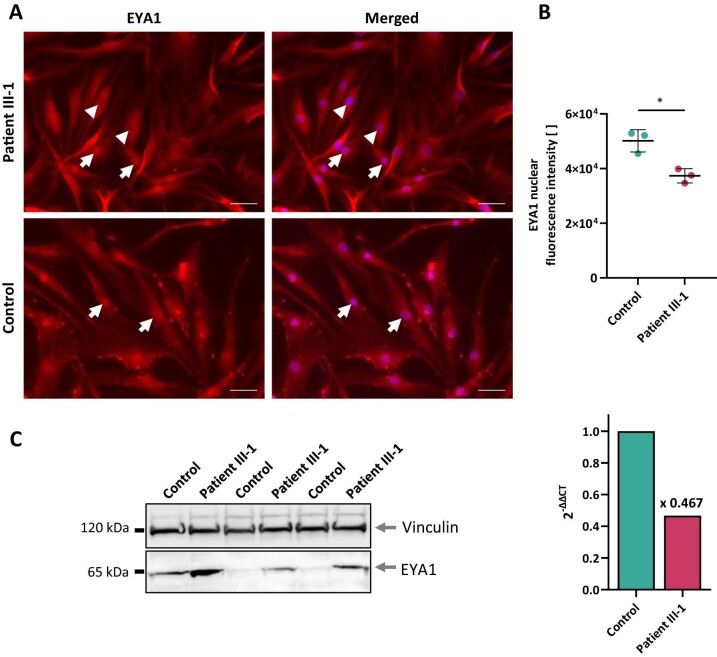
Consequences of the *EYA1* variant c.1698+1G>A for cellular EYA1 content and distribution. (A) Immunofluorescence labeling of EYA1 (red) in primary dermal fibroblasts. While control cells show consistent nuclear enrichment of EYA1 (arrows), patient cells are characterized by a heterogeneous cellular EYA1 distribution with many cells depleted of nuclear EYA1 (arrowheads). Nuclei stained with DAPI (blue). Scale bars, 50 µm. (B) Quantification of nuclear fluorescence intensities exemplified in (A). Data from three biological replicates, each averaging signal intensities from 142–263 nuclei. Unpaired two-tailed *t*-test, *p < 0.05, mean ± SD. (C) Immunoblot on whole cell lysates from primary dermal fibroblasts (left, data from three biological replicates) and qPCR data (right, mean of three technical replicates). Together, immunoblot and qPCR data corroborate the results from immunofluorescence microscopy by showing increased cytosolic EYA1 at an overall reduced *EYA1* expression level in patient cells.

To assess the functional consequences of the exon skipping at the protein level, we evaluated the intracellular EYA1 distribution pattern as well as EYA1 abundance. Immunofluorescence labeling of EYA1 in primary dermal fibroblasts consistently showed nuclear enrichment of EYA1 in control cells while many cells from patient III-1 were depleted of nuclear EYA1 (Fig. 3A). Quantification of nuclear fluorescence intensities corroborated this finding with significantly reduced fluorescence signal intensities in patient-derived cells (Fig. 3B), indicating impaired translocation of mutant EYA1 into the nucleus. Immunoblotting on whole cell lysates from dermal fibroblast cultures demonstrated increased EYA1 abundance in mutant cells, while overall *EYA1* expression was reduced upon qPCR (Fig. 3C). With our protein harvest protocol collecting the cytosolic fraction more efficiently than the nuclear one, these findings corroborate the results from immunofluorescence microscopy by showing an increase in cytosolic EYA1 in mutant fibroblasts.

### ES data analysis reveals *CYP51A1* as potential genetic modifier of renal involvement

**Figure 4:**
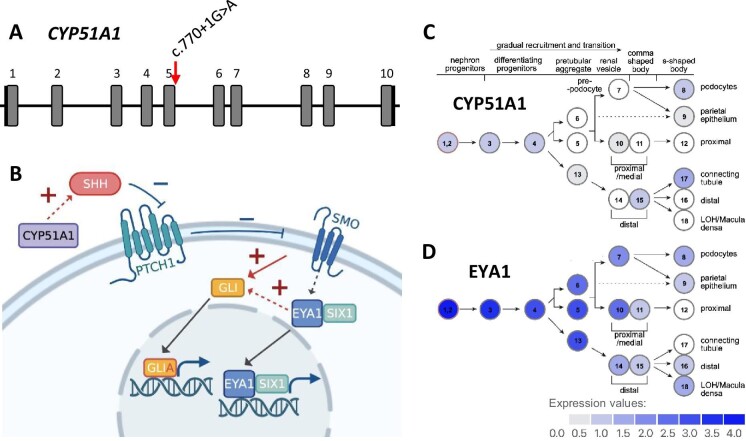
CYP51A1 plays a role in SHH signaling and is expressed in the developing kidney. (A) *CYP51A1* gene structure and location of the c.770+1G>A splice site variant (NM_000786.3). (B) Involvement of EYA1 and CYP51A1 in SHH signaling. GLI is activated into GLIA by Smoothened (SMO) which is suppressed by Patched (PTCH1). PTCH1 is removed by SHH which in turn is regulated by CYP51A1 [11]. Together with the cofactor SIX1, EYA1 enhances GLI activity [18]. This figure was created with BioRender.com. (C, D) Expression of *CYP51A1* (C) and *EYA1* (D) throughout nephron development based on single-cell RNA sequencing data from 14 weeks old human kidneys [17].

ES data from individuals III-1, III-2, IV-1, and IV-2 were analyzed for the presence of genetic variants potentially accounting for renal disease heterogeneity in BOR syndrome. Following both an unbiased approach to variant identification as well as a candidate gene approach, we identified the previously unreported *CYP51A1* variant c.770+1G>A (NM_000786.3) which was present in both children (IV-1 and IV-2) and their non-affected mother (III-2). This splice site variant is located at the splice donor of exon 5 with a predicted alteration at this site (MaxEnt: −100.0%, NNSPLICE: −100.0%), leading to an in-frame skip of exon 6 or, more likely, to an early truncation after exon 5 (Fig. 4A). It has not been listed in the gnomAD database (v2.1.1). Other candidates were not considered further due to their implausible segregation or high allele frequency (Table S1, see online supplementary material).

### Predicted CYP51A1 dysfunction does not significantly alter cholesterol intermediates in human plasma or murine kidney histology

In order to address the hypothesis that CYP51A1 affects kidney development, we analyzed mice with monoallelic *Cyp51* depletion (*Cyp51^+/^^−^*) for their kidney phenotype. The total kidney weights of *Cyp51^+/^^−^* mice were not significantly different from those of control animals. Histopathological analysis of PAS-stained kidney sections from 16 weeks old female *Cyp51^+/^^−^* (*n* = 2), male *Cyp51^+/^^−^* (n = 2), and normal control mice of both sexes (*n* = 1 per condition) did not demonstrate any macro- or microscopic abnormalities in renal tissue. Immaturity of the parenchyma, tubular cysts, inflammation, or metaplastic tissue was not observed in any of the analyzed kidneys (Fig. S2A, see online supplementary material). Additionally, glomerular size and structure did not differ between *Cyp51^+/^^−^* and control animals (Fig. S2A and B, see online supplementary material). Also, the analysis of kidney sections from both *Cyp51^−^^/^^−^* and *Cyp51^+/^^−^* mouse embryos (E14.5) did not reveal structural kidney defects (Fig. S2C, see online supplementary material).

The gene *CYP51A1* encodes the enzyme lanosterol 14α-demethylase which bears an essential role in cholesterol biosynthesis. In order to assess the impact of the identified *CYP51A1* splice site variant on enzyme function, quantitative analysis of seven main cholesterol intermediates (zymosterol, testis meiosis-activating sterol, lanosterol, 24-dehydrolathosterol, desmosterol, zymostenol, and lathosterol) was performed in the sera of three family members (III-1, III-2, IV-1) and two control subjects. These sterol intermediates were detected without any significant differences in concentrations between patient and control samples except for lanosterol (Fig. S2D, see online supplementary material). Levels of lanosterol and other sterol intermediates were slightly elevated in the non-diseased mother (III-2), likely reflecting increased synthesis rather than overt enzyme dysfunction.

In summary, *Cyp51^+/^^−^* mice were not found to develop a structural kidney phenotype and, in patient sera, a general imbalance of sterol intermediates indicative of overt CYP51A1 dysfunction was not evident. However, while these findings are in line with the notion that heterozygous *CYP51A1* depletion or dysfunction may not become apparent *per se*, they do not rule out a potential modifier role of the gene for kidney disease.

## DISCUSSION

In this study, we defined the molecular basis of BOR syndrome in a family with disease presentation in at least four generations. We identified *CYP51A1* as a potential modifier gene of renal involvement.

BOR syndrome is an exceedingly rare hereditary disease of multi organ development which manifests as branchial cysts or fistulae and affects both ear and kidney anatomy (CAKUT), leading to hearing loss and CKD, respectively [1]. Here, by clinical and genetic evaluation of participating family members, we demonstrated the broad spectrum of renal involvement within four generations of a single family segregating BOR syndrome. While the presence of preauricular pits and the development of hearing loss were very consistent throughout the generations, structural kidney disease ranged from normal kidney function in adulthood (patient III-1) to chronic kidney failure in infancy (patient IV-1).

Genetic evaluation revealed a previously reported heterozygous canonical splice site variant in *EYA1* [8], variants in which are known to account for most instances of BOR syndrome [16]. Analysis of the transcriptional effects of this splice site alteration showed aberrant *EYA1* transcripts with skipping of exon 17. These translate into EYA1 proteins which contain an alternative sequence of the highly conserved C-terminal ED. Investigation of EYA1 localization and abundance in patient-derived dermal fibroblasts showed markedly less EYA1 in the nuclear compartment as well as an increase in the cytosol. These findings are compatible with the notion that a dysfunctional ED compromises the structural association between EYA1 and co-regulators of transcription, preventing nuclear translocation of such protein complexes and impacting transcriptional regulation of target genes. Our data thus suggest that functional deletion of the ED prevents mutant EYA1 proteins from exerting their physiological role in transcriptional regulation during development. In particular, we are the first to demonstrate pathogenicity of the *EYA1* splice site variant c.1698+1G>A by *in vitro* primary patient cell analyses.

In light of the evidence for random monoallelic expression in BOR syndrome [5], we investigated the role of modifier genes as a distinct determinant of the severity of renal involvement. Analysis of ES data from two affected children, their affected father, and their non-affected mother revealed *CYP51A1* as a promising candidate gene. Its gene product lanosterol 14α-demethylase bears an essential enzymatic role in cholesterol biosynthesis. Also, it has been shown to be indispensable for embryonic development: In mice, the homozygous knock-out of *Cyp51* caused severe developmental defects and proved prenatally lethal, most likely due to defects in heart morphogenesis [11]. In the developing kidney, both *EYA1* and *CYP51A1* are expressed in nephron progenitor cells (Fig. 4C and D) [17]. Importantly, EYA1 and CYP51A1 both influence signal transduction through the SHH-dependent pathway [17, 18], a major determinant of embryonic morphogenesis (Fig. 4B) [11, 18]. After activation through binding of the transcription factor SIX1 to its highly conserved C-terminal ED, EYA1 translocates from the cytoplasm to the nucleus where it promotes gene induction of SHH pathway components [18–20]. Alterations of the SHH signaling pathway have been implicated in both developmental and degenerative instances of kidney disease, such as nephronophthisis [21, 22] and kidney fibrosis [23], supporting a role for disease modification [24]. A specific example is Pallister-Hall syndrome (MIM #146510) where genetic alterations of GLI3, a transcriptional effector in the SHH signaling pathway, lead to developmental defects of multiple organ systems including uni- and bilateral CAKUT [25].

*CYP51A1* variants are rare [26, 27] and have not previously been associated with kidney disease. In line with this, our histopathological analysis did not show significant structural alterations in kidneys from *Cyp51^+/^^−^* or *Cyp51^−^^/^^−^* mice when compared to control animals. Also, measurements of sterol intermediates in serum samples from three family members did not indicate dysfunction of the CYP51A1 enzyme under baseline conditions. Of note, inactivation of both *Cyp51* alleles in the murine liver led to marked increases (between 30$\times $ and more than 300$\times $) in tissue lanosterol and dihydrolanosterol levels [12, 28], whereas in the *Cyp51^+/^^−^* mouse embryos, lanosterol and dihydrolanosterol were elevated by 3–6-fold [11]. Thus, monoallelic defects in *CYP51A1* may not be expected to significantly impact cholesterol biosynthesis in human subjects.

While our data indicate that the *CYP51A1* variant identified here does not impact CYP51A1 function in a disease-causing manner, its potential role as a modifier during nephrogenesis cannot be ruled out. Given the roles EYA1 and CYP51A1 play in development and, in particular, in modulating the SHH pathway, further research is needed to illuminate their mutual interference in regulating transcription throughout development.

In conclusion, our data indicate that *EYA1* c.1698+1G>A proves pathogenic by functional deletion of the ED, preventing EYA1 from exerting its role in transcriptional regulation during development. Random monoallelic expression was proposed to underly the marked phenotypic variability in BOR syndrome [5]. In addition, we here suggest *CYP51A1* to potentially play a role as genetic modifier of renal involvement in BOR syndrome. While our data indicate that the heterozygous knock-out of *CYP51A1* is not sufficient to cause kidney disease, we conjecture that defective CYP51A1 aggravates renal phenotypes by adding to the disruptive effect of disease-causing *EYA1* mutations on SHH-dependent signal transduction throughout renal development. Comprehensive genetic testing of further families affected by BOR syndrome will be crucial to determine the significance of this hypothesis.

## Supplementary Material

sfad260_Supplemental_File

## Data Availability

The data that support the findings of this study are available from the corresponding author upon reasonable request.
