# Supplementary material for: Mechanisms of pathogenicity and the quest for genetic modifiers of kidney disease in branchiootorenal syndrome
Source: Clin Kidney J. 2023 Oct 13;17(1):sfad260. doi: 10.1093/ckj/sfad260 (PMC10783239; doi:10.1093/ckj/sfad260)
Supplement: sfad260_Supplemental_File [file sfad260_supplemental_file.pdf]

A

Exon 16
GACAAACTGTGTGAATATTTTAGTAACAACTACTCAGCTCATCCCAGCATTG
GCGAAAGTCCTGCTGTATGGGTTAGGAATTGTATTTCCAATAGAAAATATTT
ACAGTGCAACTAAAATAG

Exon 17
GAAAAGAAAGCTGTTTTGAGAGAATAATTCAAAGGTTTGGAAGAAAAGTGGT
GTATGTTGTTATAGGAGATGGTGTAGAAGAAGAACAAGGAGCAAAAAAG

c.1698

Exon 18
CACGCGATGCCCTTCTGGAGGATCTCCAGCCACTCGGACCTCATGGCCCTGC
ACCACGCCTTGGAACTGGAGTACCTGTAACAGCGCTCGGCACTTTGACAGCG
CACAG...

B

ERVFIWDLDETIIVFHSLLTGSYANRYGRDPPTSVSLGLRMEEMIFNLADTH
LFFNDLEECDQVHIDDVSSDDNGQDLSTYNFGTDGFPAAATSANLCLATGVR
GGVDWMRKLAFRYRRVKEIYNTYKNNVGGLLGPAKREAWLQLRAEIEALTDS
WLTLALKALSLIHSRTNCVNILVTTTQLIPALAKVLLYGLGIVFPIENIYSA
TKIGKESCFERIIQRFGRKVVYVVIGDGVEEEQGAKKHAMPFWRISSHSDLM
ALHHALELEYL

ERVFIWDLDETIIVFHSLLTGSYANRYGRDPPTSVSLGLRMEEMIFNLADTH
LFFNDLEECDQVHIDDVSSDDNGQDLSTYNFGTDGFPAAATSANLCLATGVR
GGVDWMRKLAFRYRRVKEIYNTYKNNVGGLLGPAKREAWLQLRAEIEALTDS
WLTLALKALSLIHSRTNCVNILVTTTQLIPALAKVLLYGLGIVFPIENIYSA
TKIARDALLEDLQPLGPHGPAPRLGTGVPVTALGTLTAHSCSVTRDRSSRPQ
SRISAGLQNLAISAW

C

**Human**	ERVFIWDLDETIIVFHSLLTGSYANRYGRDPPTSVSLGLRMEE
**Chimpanzee**	ERVFIWDLDETIIVFHSLLTGSYANRYGRDPPTSVSLGLRMEE
**Mouse**	ERVFIWDLDETIIVFHSLLTGSYANRYGRDPPTSVSLGLRMEE
**Zebrafish**	ERVFIWDLDETIIVFHSLLTGSYANRFGRDPPTSVSLGLRMEE
**Human**	MIFNLADTHLFFNDLEECDQVHIDDVSSDDNGQDLSTYNFGTD
**Chimpanzee**	MIFNLADTHLFFNDLEECDQVHIDDVSSDDNGQDLSTYNFGTD
**Mouse**	MIFNLADTHLFFNDLEECDQVHIDDVSSDDNGQDLSTYNFGTD
**Zebrafish**	MIFNLADTHFFFNDLEECDQVHIDDVSSDDNGQDLSTYNFSTD
**Human**	GFPAAATSANLCLATGVRGGVDWMRKLAFRYRRVKEIYNTYKN
**Chimpanzee**	GFPAAATSANLCLATGVRGGVDWMRKLAFRYRRVKEIYNTYKN
**Mouse**	GFPAAATSANLCLATGVRGGVDWMRKLAFRYRRVKEIYNTYKN
**Zebrafish**	GFHAAATSANLCLATGVRGGVDWMRKLAFRYRRVKEIYTTYKN
**Human**	NVGGLLGPAKREAWLQLRAEIEALTDSWLTLALKALSLIHSRT
**Chimpanzee**	NVGGLLGPAKREAWLQLRAEIEALTDSWLTLALKALSLIHSRT
**Mouse**	NVGGLLGPAKREAWLQLRAEIEALTDSWLTLALKALSLIHSRT
**Zebrafish**	NVGGLLGPAKREAWLQLRAEIEALTDSWLTLALKALTLIHSRS
**Human**	NCVNILVTTTQLIPALAKVLLYGLGIVFPIENIYSATKIGKES
**Chimpanzee**	NCVNILVTTTQLIPALAKVLLYGLGIVFPIENIYSATKIGKES
**Mouse**	NCVNILVTTTQLIPALAKVLLYGLGIVFPIENIYSATKIGKES
**Zebrafish**	NCVNILVTTTQLIPALAKVLLYGLGVVFPIENIYSATKIGKES
**Human**	CFERIIQRFGRKVVYVVIGDGVEEEQGAKKHAMPFWRISSHSD
**Chimpanzee**	CFERIIQRFGRKVVYVVIGDGVEEEQGAKKHAMPFWRISSHSD
**Mouse**	CFERIIQRFGRKVVYVVIGDGVEEEQGAKKHAMPFWRVSSHSD
**Zebrafish**	CFERVIQRFGRKVVYVVVGDGVEEEQGSKKHNMPFWRISSHSD
**Human**	LMALHHALELEYL
**Chimpanzee**	LMALHHALELEYL
**Mouse**	LMALHHALELEYL
**Zebrafish**	LMALHHALDLEYL

Figure S1. (A) cDNA sequence of the mutant *EYA1* transcript corresponding to the lower band in the middle lane of the gel image depicted in Figure 2C. The sequence shows the skip of exon 17 (highlighted in red) and the ensuing frameshift as a consequence of c.1698+1G>A. Sanger sequencing also revealed c.1755T>C which is highlighted in yellow. Grey, not sequenced; blue, protein-coding bases covered by Sanger sequencing; orange, part of 3' untranslated region covered by Sanger sequencing. For reverse transcription, RNA was extracted from dermal fibroblasts derived from patient III-1. (B) Wild-type (top) and predicted mutant (bottom) EYA domain protein sequences based on NP_000494.2, showing frameshift-induced aberrant C-terminal residues (red vs. green letters). As a consequence of c.1755T>C, the position highlighted in yellow harbors an arginine instead of a cysteine residue. (C) Alignment of EYA domain protein sequences (EYA1 amino acid positions 322-592) from different species, demonstrating high conservation of the protein domain. Red boxes highlight the residues affected by c.1698+1G>A. Blue and black letters mark consecutive exons, respectively, and red letters indicate residues which overlap splice sites.

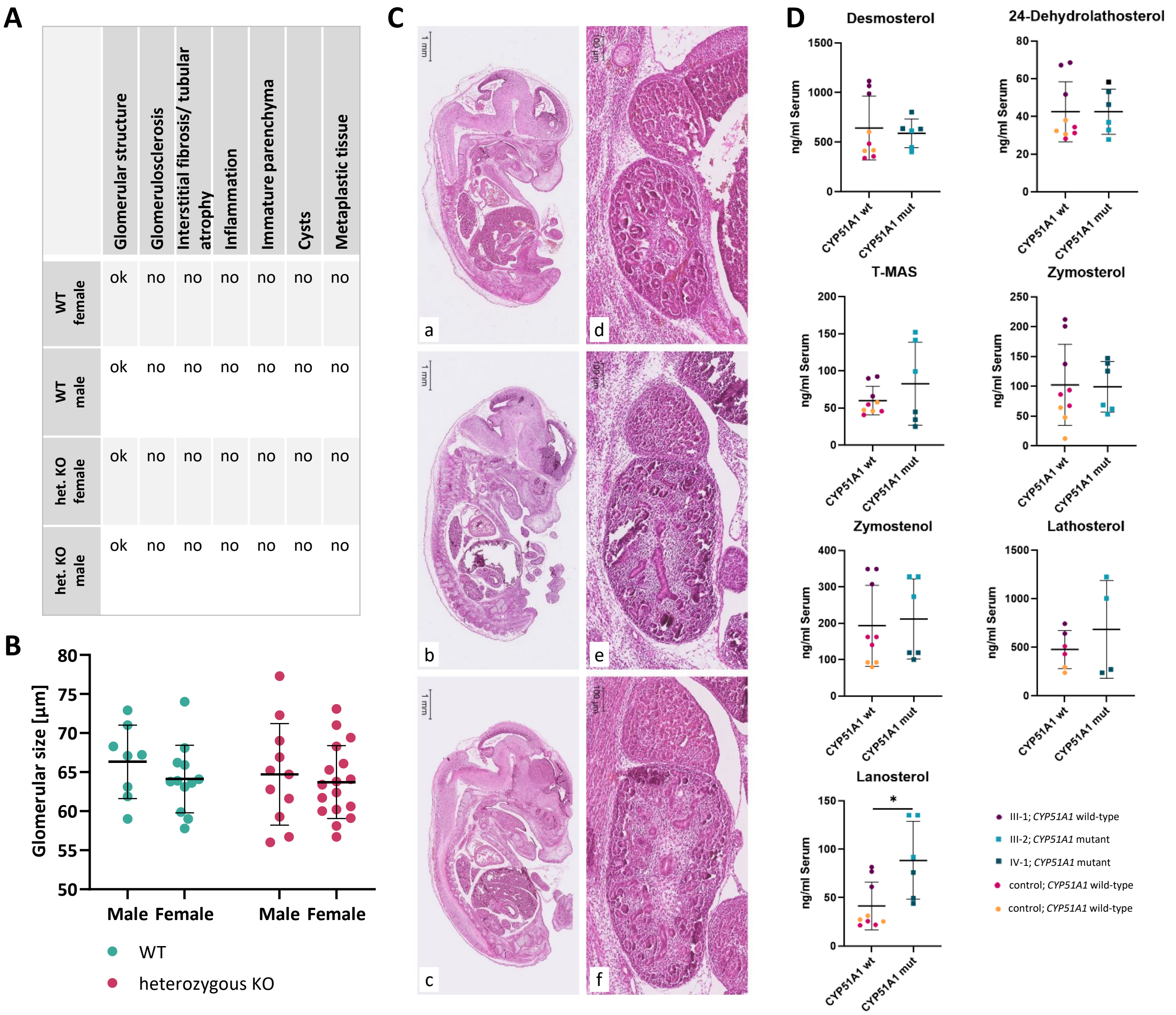

Figure S2. Histopathological analysis of kidneys from *Cyp51*$^{+/-}$ and *Cyp51*$^{-/-}$ mice and measurement of sterol intermediates in patient sera. (A) Histopathological analysis of kidney sections from *Cyp51*$^{+/-}$ (heterozygous knock-out) and wild-type mice did not show any significant differences with respect to glomerular structure, glomerulosclerosis, interstitial fibrosis/ tubular atrophy, inflammation, immature parenchyma, cysts, or metaplastic tissue. (B) Glomerular sizes did not significantly differ between kidneys from *Cyp51*$^{+/-}$ mice and those from wild-type animals. Two-way ANOVA, mean ± SD. (C) For *Cyp51*$^{-/-}$ (a,d), *Cyp51*$^{+/-}$ (b,e), and wild-type (c,f) mouse embryos at E14.5, representative sagittal sections (left) are shown alongside higher magnifications of the kidneys (right). Normal renal development with primitive glomeruli in the cortex as well as collecting ducts and stromal cells in the medulla are present in all three genotypes. (D) Quantitative analysis of seven main cholesterol intermediates in three family members (III-1, *CYP51A1* wild-type, violet; III-2, *CYP51A1* mutant, light turquoise; IV-1, *CYP51A1* mutant, dark turquoise) and two control subjects (*CYP51A1* wild-type, pink and orange, respectively) with three technical replicates each. The measurements were not significantly different (p>0.7) between samples harboring wild-type and mutant *CYP51A1* except for the lanosterol analysis (p=0.0256). Mann Whitney tests, mean ± SD. het., heterozygous; KO, knock-out; WT/ wt, wild-type; mut, mutant, i.e., harboring *CYP51A1* c.770+1G>A in a heterozygous fashion; T-MAS, testis meiosis-activating sterol.

Gene	Variant	Poly-Phen-2	PROVEAN	MaxEnt, NNSPLICE	Allele frequency*	IV-1	IV-2	III-1	III-2
CYP51A1	c.770+1G>A p.?	n/a	n/a	-100.0% -100.0%	not known	het	het		het
LRRD1	c.1419dup p.(Asn474*)	n/a	deleterious	no predicted impact	not known		het	het	
SPAG9	c.2081A>G p.(Asn694Ser)	possibly damaging	deleterious	no predicted impact	0.0002402	het	het		het

Table S1. Candidate genes from exome sequencing identified in family members IV-1, IV-2, III-1, or III-2. *LRRD1* and *SPAG9* were not considered further due to their implausible segregation or high allele frequency, respectively. n/a, not available; het, heterozygous; * gnomAD database (European, non-Finnish population).
